# Influence of Enthusiastic Language on the Credibility of Health Information and the Trustworthiness of Science Communicators: Insights From a Between-Subject Web-Based Experiment

**DOI:** 10.2196/13619

**Published:** 2019-08-12

**Authors:** Lars König, Regina Jucks

**Affiliations:** 1 Department of Psychology University of Münster Münster Germany

**Keywords:** health communication, information seeking behavior, trust, language, occupations, deep learning, FMRI, source credibility, persuasiveness

## Abstract

**Background:**

To decide whether online health information is reliable, information seekers apply 2 stretegies: first, information seekers can make credibility judgments by using their prior knowledge to evaluate the validity of the encountered health claim. Second, instead of evaluating the health claim itself, information seekers can make trustworthiness judgments by evaluating the character of the information source. In recent years, information givers from various professions have begun to use enthusiastic language to disseminate their information and persuade their audiences.

**Objective:**

To systematically explore this phenomenon, the goal of this study was to answer the following research questions: (1) does an enthusiastic language style, in comparison with a neutral language style, increase the trustworthiness of a person arguing in an online health forum and the credibility of his or her information? (2) does working for a university, in comparison with working for a lobbying organization, increase the trustworthiness of a person arguing in an online health forum and the credibility of his or her information? (3) does working for a university in combination with using an enthusiastic language style result in especially high trustworthiness and credibility ratings?

**Methods:**

In a 2x2 between-subject online experiment, 270 participants read a post from an online health forum and subsequently rated the trustworthiness of the forum post author and the credibility of his information. A total of 2 aspects of the forum post varied, namely the professional affiliation of the forum post author (whether the person introduced himself as a scientist or a lobbyist) and his language style (whether he used a neutral language style or an enthusiastic language style).

**Results:**

When the forum post author used an enthusiastic language style, he was perceived as more manipulative (*P*<.001), less knowledgeable (*P*<.001), and his information was perceived as less credible (*P*<.001). Overall, scientists were perceived as less manipulative (*P*=.04) than lobbyists. Furthermore, language style and professional affiliation interacted: When the forum post author was a lobbyist, language style did not affect integrity (*P*=.96) and benevolence (*P*=.79) ratings. However, when the forum post author was a scientist, enthusiastic language led to lower integrity (*P*=.002) and benevolence (*P*<.001) ratings than neutral language.

**Conclusions:**

The current findings illustrate that health information seekers do not just react to online health information itself. In addition, they are also sensitive to the ways in which health information is presented (“Which langue style is used to communicate health information?”) and who presents it (“Who does the health information source work for?”).

## Introduction

### Background

How do information seekers decide whether they can rely on online health information? The importance of this question is stressed by 2 recent developments: First, information seekers have developed diverse ways to acquire online health information [1], and they rely on it frequently [2]. Second, online health information often contains misinformation [3-5] because the internet is not governed by professional editors [6,7]. Hence, information seekers constantly have to decide whether they should rely on the health information they encounter online. According to the content-source integration model [8], 2 strategies can be applied to make such decisions. First, information seekers can make credibility judgments (first-hand evaluations) by using their prior knowledge to evaluate the validity of the encountered health information claim. Second, instead of evaluating the health information claim itself, information seekers can make trustworthiness judgments (second-hand evaluations) by evaluating the character of the information source. As most information seekers have just a bounded understanding of science, they often lack the necessary expertise to make accurate credibility judgments [9,10]. Therefore, information seekers frequently have to turn to experts and evaluate their trustworthiness [11-14]. This development gives rise to another intriguing question: Which factors influence information seekers’ credibility and trustworthiness judgments?

### Language Styles and Their Relationship to Credibility and Trustworthiness

Various factors influence information seekers’ credibility and trustworthiness judgments [15-17], but the language style of an information source seems to be an especially influential factor [18-22]. Thon and Jucks [18], for example, showed that the authors of health information forum posts were rated as more trustworthy and their information as more credible when they used an everyday language style (eg, “heart attack”) instead of a technical language style (eg, “myocardial infarction”). Furthermore, Mayweg-Paus and Jucks [22] showed that participants accepted information from an online health article to a higher degree and processed it in more depth when the article was written in a tentative language style (eg, “is presumably similar”) rather than a nontentative language style (eg, “is similar”). Tentativeness, however, does not just infuence trustworthiness and credibility judgments. In a study by Feinkohl et al [23], participants saw an online news article about the therapeutic application of deep brain stimulation. Depending on the experimental condition, the article was accompanied by different online forum comments or no comments at all. The results showed that participants were more likely to address the tentativeness of the findings in their own forum comments if they had previously seen forum comments that addressed this issue too (for additional information on scientific tentativeness and medical research, see the study by Flemming et al [24]). In another study, König and Jucks [20] showed that science communicators in scientific health debates were rated as more trustworthy and their information as more credible when they used a neutral language style (eg, “a series of methodological mistakes”) instead of an aggressive language style (eg, “a series of really dumb methodological mistakes”). Besides influencing trustworthiness and credibility judgments, the use of emotional language also influences interactions in online forums and risk perceptions. For example, research showed that medical students incease their emotional language use when replying to emotional patient queries in medical forums [25]. Furthermore, a recent study showed that emotionalization can influence risk perceptions in a science communication context [26]. Aggressive, technical, tentative, and everyday language styles, however, are not the only language styles that are used to disseminate health information.

### This Study: How Does Enthusiastic Language Influence the Credibility of Health Information and the Trustworthiness of Science Communicators?

In recent years, more and more people use enthusiastic language to disseminate information and persuade their audiences in diverse contexts. For example, Ghose et al [27] observed that, instead of just writing positive comments on online marketplace platforms, “buyers tend to use superlatives and highly enthusiastic language to praise a good merchant.” In another study, Barry et al [28] pointed out that enthusiastic language is used as a persuasion tactic on commercial websites. Furthermore, enthusiastic language is nowadays often used to communicate scientific findings. Instead of using standard academic language, authors of scientific articles increasingly express their enthusiasm about their findings by including enthusiastic phrases in their article titles and texts, for example, “Fantastic beasts” [29] and “The Incredible Shrinking Spindle” [30].

So far, it is not clear why so many people use enthusiastic language when communicating information. One reason might be that enthusiasm is often encouraged in educational settings because it has been linked to various positive outcomes [31]. For example, teacher enthusiasm is linked to students’ enjoyment [32], interest [33], achievement [34], motivation, and vitality [35]. Therefore, information givers might think that using enthusiastic language to communicate information makes them especially effective and follows best-practice examples. But is this true? Interestingly, no research so far has systematically explored whether enthusiastic language influences the trustworthiness of an information source and the credibility of his or her information. And even if it does, would using enthusiastic language be equally effective for people from different professions? Burgoon et al [36], for example, argue in the context of Language Expectancy Theory that “highly credible communicators have the freedom (wide bandwidth) to select varied language strategies and compliance-gaining techniques in developing persuasive messages, whereas low-credible communicators must conform to more limited language options if they wish to be effective.” Following this argumentation, one would expect that high-credibility communicators such as scientists would benefit more from the use of an enthusiastic language style than low-credibility communicators such as lobbyists. To test these hypotheses, we developed a between-subject online experiment. In the experiment, participants read a post from an online health forum and subsequently rated the trustworthiness of the forum post author and the credibility of his information. We chose online health forums because they typically allow users from diverse educational and professional backgrounds to exchange information in an unrestricted way. Furthermore, diverse research findings suggest that information seekers frequently rely on online forums to aquire health information [37-40]. Within the forum post, we varied the professional affiliation of the forum post author (whether the person introduced himself as a scientist or a lobbyist) and his language style (whether he used a neutral language style or an enthusiastic language style). The goal was to test the following hypotheses:

Hypothesis 1: An enthustiastic language style, in comparision with a neutral language style, increases the trustworthiness of a person arguing in an online health forum and the credibility of his information.

Hypothesis 2: Working for a university, in comparison with working for a lobbying organization, increases the trustworthiness of a person arguing in an online health forum and the credibility of his information.

Hypothesis 3: Working for a university in combination with using an enthusiastic language style results in especially high trustworthiness and credibility ratings.

## Methods

### Design and Material

We used a 2 (language style: neutral language vs enthusiastic language) x 2 (professional affiliation: scientist vs lobbyist) between-subject experimental design, resulting in 4 experimental conditions. In each experimental condition, participants saw 2 online forum posts: a question post and an answer post. The 2 posts were embedded in a screenshot of a website. The URL of the website indicated that it was an online health forum. In the question post, a woman stated that there currently is a controversial debate about whether functional magnetic resonance imaging and artificial intelligence technologies can be combined to improve medical diagnoses. Following this, she asked whether anybody could explain the technologies to her. The question post was written in a neutral language style and was the same in all 4 experimental conditions. Textbox 1 shows the text of the question post. The textbox shows an English translation of the German post. Therefore, the translated version may not appear as authentic to native English speakers as the original version appears to native German speakers. The original German version of the question post can be obtained from the authors upon request. In the answer post, a man introduced himself and explained the basics of the functional magnetic resonance imaging technology. Following this, he described the results of a study [41] that had combined functional magnetic resonance imaging and artificial intelligence technologies. The experimental manipulations were realized in the answer post. Depending on the experimental condition, the answer post was written either in (1) a neutral language style or (2) an enthusiastic language style. Furthermore, the author of the answer post introduced himself either as (1) a scientist who worked for an imaging technology institute at a university or (2) a lobbyist who worked at an imaging technology lobbying organization. Textbox 2 shows the text of the answer post with the experimental manipulation. The enthusiastic language style version of the answer post contained the words and phrases printed in italics and the neutral language style version did not contain these words and phrases. The textbox shows an English translation of the German post. Therefore, the translated version may not appear as authentic to native English speakers as the original version appears to native German speakers. The original German version of the post can be obtained from the authors upon request.

**Text of the question post.**

Dear forum community,

Functional magnetic resonance imaging allows activated brain areas to be spatially displayed. Increasingly, this technology is combined with methods of artificial intelligence, creating new applications. There is currently a lot of controversy as to whether the emerging methods should be used to diagnose illnesses (such as depression).

The opinions are very different. Advocates argue that diagnoses would become clearer through the use of the new methods. Critics argue that the potential of the new methods is overestimated and could lead to devastating misdiagnoses.

Does anyone know this technology and can give me a brief introduction?

Thanks in advance!

Sabine Schneider

**Text of the answer post with the experimental manipulation.**

Hello Mrs. Schneider,

[Scientist Manipulation:] My name is Johannes Becker and I work for the Institute for Imaging Technology at the University of Bochum.

[Lobbyist Manipulation:] My name is Johannes Becker and I work for the Association of Imaging Technology-Producing Industries in Bochum.

I can give you a brief insight into the functionalities and applications of functional magnetic resonance imaging. *And what I can tell you at the beginning: I think the topic is fascinating!*

First to the basics: How does functional magnetic resonance imaging work? Nerve cells require more nutrients when active than at rest. If a particular brain region is active, the metabolism in this region increases. In response to this increased metabolism, the body transports more oxygen-rich blood into the activated brain region. Functional magnetic resonance imaging measures these changes in blood flow and uses them as an indicator of activity in different brain regions. *In my opinion, this is a simple and ingenious method!*

Now to the possible applications: The possible applications of functional magnetic resonance imaging are manifold *and I personally think many of them are breathtaking*. A current example of use *- that absolutely excites me -* is this:

Researchers from the US have combined functional magnetic resonance imaging and deep-learning technologies to decipher various activities of the human brain. For this purpose, the researchers showed their participants a series of videos and simultaneously recorded their brain activity using functional magnetic resonance imaging. The collected data was then fed to algorithms and they learned how the video clips and the brain activities were related to each other.

Afterwards, the researchers examined what the algorithms were capable of and in the following I would like to focus briefly on three central results *that astonish me again and again.*

The first result: the researchers provided one of the algorithms with new videos, and the algorithm was able to predict what brain activity the new videos would trigger in the participants. *I think this result is simply groundbreaking!*

The second result *- which I find very interesting -* is the following: One of the algorithms could later read from the brain activities of the participants, what the participants saw at a certain moment. For example, the algorithm could say with high accuracy whether the subjects were seeing an airplane, a bird, or a face.

Now the third result *and this result amazes me again and again when I talk about it:* One of the algorithms was able to draw a picture of what the participants saw at certain moments. The images had not the best resolution, but you could see the outlines and contours.

I hope you learned something new about the functionalities and applications of functional magnetic resonance imaging *and who knows, maybe you are now just as excited as I am about this topic.*

Yours sincerely,

Johannes Becker

### Procedure

The experiment was conducted online using the Questback Enterprise Feedback Suite Survey platform for data collection. Before the experiment started, participants were told that the experiment would address the communication of information in online forums. Furthermore, they were informed about the general procedure of the upcoming experiment and that they could end the experiment at any time. To start the experiment, participants had to indicate that they had read all provided information and that they agreed to take part in the experiment. On the remaining pages, participants indicated their age, gender, whether they studied at the bachelor’s or master’s level, the university where they studied, their study subjects, and the semester they were currently in. Furthermore, they answered the control measures (see section “Control Measures”). Following this, participants were randomly assigned to 1 of the 4 experimental conditions and were presented with the corresponding online forum posts (see section “Design and Material”). After reading the forum posts, participants answered the dependent measures (see section “Dependent Measures”). After answering the dependent measures, participants answered the manipulation check question (see section “Manipulation Check”) and were debriefed. They were told about the manipulations of the experiment and that they could contact the leading scientist if they had any further questions or comments. Furthermore, they could choose to leave their information to get reimbursed for their participation. The experiment was designed to comply with the ethical guidelines developed by the American Psychological Association and the German Psychological Society.

### Sample

German university students enrolled in diverse majors from the humanities and sciences were contacted via email and social network sites and received 5 Euro for participating in the online experiment. Participants who indicated at the end of the study that they answered the questions honestly and completed the study without interruption and technical problems were included in data analyses. The sample contained 270 (207 female, 63 male) students (149 undergraduate students, 121 graduate students) with an average age of 23 years (mean 23.39, SD 3.04). Furthermore, the average participant was enrolled in their study program for 7 semesters (mean 6.73, SD 3.96) and took 12 min (mean 11.51, SD 5.09) to complete the study.

### Control Measures

A total of 3 control measures were included to assess whether the experimental groups differed in regard to characteristics that could affect the study results. It is possible that people who frequently use online forums are better able to identify the quality of online forum posts and their content than people who just occasionally use online forums. Therefore, participants answered the questions “How often do you visit Internet forums?” (general use) and “How often do you visit Internet forums to learn something new or acquire new skills?” (educational use) on a scale ranging from 1 (very rarely) to 7 (very often). Furthermore, it is possible that people who are well informed about a topic make different credibility and trustworthiness judgments than people who are less informed about the same topic. Therefore, participants answered the question “How much do you know about functional magnetic resonance imaging and deep learning?” (prior knowledge) on a scale ranging from 1 (very little) to 7 (very much).

### Manipulation Check

To check whether the language style manipulation was successful, participants answered the question “How would you describe Johannes Becker’s choice of words?” on a scale ranging from 1 (neutral) to 7 (extremely enthusiastic). In addition, we assessed the strength of the experimental manipulations by asking the participants whether they remembered the language style and professional affiliation of the answer post author. To assess whether the participants remembered the language style, they were asked whether certain enthusiastic expressions were used in the answer post. Participants could chose between “Yes,” “No,” and “I do not know.” To assess whether the participants remembered the professional affiliation of the answer post author, they were asked “For whom did Johannes Becker work?” Participants could choose between “Institute for Imaging Technology at the University of Bochum,” “Association of Imaging Technology-Producing Industries in Bochum,” and “I do not know.”

### Dependent Measures

For each dependent measure, a total score was generated by calculating the mean.

#### Message Credibility

As a credibility measure of the provided information, the Message Credibility Scale [42] was translated and adapted. Participants indicated their agreement with 3 statements, for example, “The provided information was believable.”

#### Machiavellianism

The German version of the Machiavellianism Subscale from the Dirty Dozen Scale [43,44] was used to assess how manipulative the forum post author was perceived to be. Participants indicated their agreement with 4 statements, for example, “Johannes Becker has used deceit or lied to get his way,” on a scale ranging from 1 (totally disagree) to 7 (totally agree).

#### Expertise, Integrity, and Benevolence

The Muenster Epistemic Trustworthiness Inventory [45] was used to assess how trustworthy the forum post author was perceived to be. Participants rated 15 items on a scale ranging from 1 (not trustworthy at all) to 7 (very trustworthy). The items measured expertise (eg, “unqualified-qualified”), benevolence (eg, “immoral-moral”), and integrity (eg, “insincere-sincere”).

## Results

### General Procedure

For all analyses, the statistical software IBM SPSS Statistics version 25 was used. For the analyses of the dependent measures, 2-way between-subject analyses of variance were conducted with language style (neutral language vs enthusiastic language) and professional affiliation (scientist vs lobbyist) as independent variables. Type 3 sum of squares were used. For all analyses, the alpha level was set at .05. The dataset contains further variables that have not been described in this study and have not been analyzed yet because they exceed the scope of this study.

### Control Measures

Before analyzing the dependent measures, 4 1-way between-subject analyses of variance were conducted with experimental condition as the independent variable and the control measures as dependent variables to analyze whether the participants in the 4 experimental groups differed in aspects that could influence the study results. The results showed that the participants in the 4 experimental groups did not significantly differ in regard to their general online forum use (general use: *F*_3,266_=0.817; *P*=.49), online forum use for educational purposes (educational use: *F*_3,266_=0.052; *P*=.98), and their prior knowledge (prior knowledge: *F*_3,266_=1.071; *P*=.36). Therefore, the 3 control measures were not included in further analyses.

### Manipulation Check

Participants in the enthusiastic language style condition (mean 6.04, SD 0.95) perceived the choice of words as more enthusiastic than participants in the neutral language style condition (mean 2.37, SD 1.26); *t*_244.992_=-26.890; *P*<.001. Hence, the language style manipulation worked as expected. Furthermore, 252 (93%) participants correctly remembered the language style of the answer post author, and 231 (86%) correctly remembered his professional affiliation. A total of 216 (80%) participants remembered both correctly. The high remembrance rates suggest that the vast majority of participants recognized the experimental manipulations. As information seekers naturally differ in their attention to detail in real-world settings and the experimental manipulations might not need to be consciously remembered to have an effect, we included all participants in the data analyses.

### Dependent Measures

Tables 1 and 2 show the means and SDs of the dependent measures.

**Table 1:** Main effects: means and SDs of the dependent measures by language style and professional affiliation.

Dependent measures^a^		Language style				Professional affiliation		
		Neutral (n=133), mean (SD)	Enthusiastic (n=137), mean (SD)	*P* value	Scientist (n=135), mean (SD)		Lobbyist (n=135), mean (SD)	*P* value
**Credibility**								
	Message credibility	5.11 (1.04)	4.48 (1.23)	<.001	4.83 (1.16)		4.75 (1.19)	.56
**Trustworthiness**								
	Machiavellianism	2.70 (1.17)	3.85 (1.23)	<.001	3.13 (1.28)		3.44 (1.37)	.04
	Expertise	5.44 (0.98)	4.90 (1.19)	<.001	5.15 (1.11)		5.19 (1.13)	.75
	Integrity^b^	4.67 (0.91)	4.42 (1.03)	.03	4.54 (0.92)		4.55 (1.04)	.95
	Benevolence^b^	4.40 (0.82)	4.10 (1.02)	.006	4.21 (0.94)		4.29 (0.93)	.52

^a^General interpretation: On the Machiavellianism scale, a low score indicates high trustworthiness and a high score indicates low trustworthiness. On all other scales, a low score indicates low trustworthiness/credibility and a high score indicates high trustworthiness/credibility. All scales ranged from 1 to 7.

^b^Interaction is significant; see Table 2.

**Table 2:** Interaction effects: means and SDs of the dependent measures by language style and professional affiliation.

Dependent measures^a^	Scientist			Lobbyist		
	Neutral (n=67), mean (SD)	Enthusiastic (n=68), mean (SD)	*P* value	Neutral (n=66), mean (SD)	Enthusiastic (n=69), mean (SD)	*P* value
Integrity	4.79 (0.87)	4.28 (0.90)	.002	4.54 (0.94)	4.55 (1.13)	.96
Benevolence	4.50 (0.83)	3.93 (0.96)	<.001	4.31 (0.79)	4.26 (1.05)	.79

^a^On all scales, a low score indicates low trustworthiness and a high score indicates high trustworthiness. All scales ranged from 1 to 7.

#### Message Credibility

There was a significant main effect of language style (*F*_1,266_=20.300; *P*<.001; η_p_^2^=.071) on message credibility, indicating that enthusiastic language led to lower message credibility ratings than neutral language. There was no main effect of professional affiliation (*F*_1,266_=0.334; *P*=.56; η_p_^2^=.001) and no interaction effect (*F*_1,266_=0.178; *P*=.67; η_p_^2^=.001).

#### Machiavellianism

There was a significant main effect of language style (*F*_1,266_=62.898; *P*<.001; η_p_^2^=.191) on Machiavellianism, indicating that enthusiastic language led to higher Machiavellianism ratings than neutral language. Furthermore, there was a significant main effect of professional affiliation (*F*_1,266_=4.487; *P*=.04; η_p_^2^=.017) on Machiavellianism, indicating that being a lobbyist led to higher Machiavellianism ratings than being a scientist. There was no interaction effect (*F*_1,266_=0.010; *P*=.92; η_p_^2^<.001).

#### Expertise

There was a significant main effect of language style (*F*_1,266_=16.357; *P*<.001; η_p_^2^=.058) on expertise, indicating that enthusiastic language led to lower expertise ratings than neutral language. There was no main effect of professional affiliation (*F*_1,266_=0.106; *P*=.75; η_p_^2^<.001) on expertise and no interaction effect (*F*_1,266_=0.008; *P*=.93; η_p_^2^<.001).

#### Integrity

There was a significant main effect of language style (*F*_1,266_=4.530; *P*=.03; η_p_^2^=.017) on integrity, indicating that enthusiastic language led to lower integrity ratings than neutral language. There was no main effect of professional affiliation (*F*_1,266_=0.004; *P*=.95; η_p_^2^<.001) on integrity. However, the interaction was significant (*F*_1,266_=4.863; *P*=.03; η_p_^2^=.018). Simple effect analysis indicated that when the forum post author was a lobbyist, language style did not affect integrity (*F*_1,266_=0.003; *P*=.96; η_p_^2^<.001). However, when the forum post author was a scientist, enthusiastic language led to lower integrity ratings than neutral language (*F*_1,266_=9.392; *P*=.002; η_p_^2^=.034).

#### Benevolence

There was a significant main effect of language style (*F*_1,266_=7.633; *P*=.006; η_p_^2^=.028) on benevolence, indicating that enthusiastic language led to lower benevolence ratings than neutral language. There was no main effect of professional affiliation (*F* 1_,266_=0.422; *P*=.52; η_p_^2^=.002) on benevolence. However, the interaction was significant (*F*_1,266_=5.679; *P*=.02; η_p_^2^=.021). Simple effect analysis indicated that when the forum post author was a lobbyist, language style did not affect benevolence (*F*_1,266_=0.072; *P*=.79; η_p_^2^<.001). However, when the forum post author was a scientist, enthusiastic language led to lower benevolence ratings than neutral language (*F*_1,266_=13.243; *P*<.001; η_p_^2^=.047).

## Discussion

### Principal Findings

A total of 3 hypotheses addressed the effects of language style (neutral vs enthusiastic) and professional affiliation (scientist vs lobbyist) in online health forums. We hypothesized that an enthusiastic language style, in comparison with a neutral language style, would positively affect the trustworthiness of a forum post author and the credibility of his information. Contrary to our hypothesis, we found that when the forum post author used an enthusiastic language style, he received higher Machiavellianism ratings, lower expertise ratings, and lower message credibility ratings. Furthermore, we hypothesized that working for a university, in comparison with working for a lobbying organization, would positively affect the trustworthiness of a forum post author and the credibility of his information. The results partly confirm this hypothesis: Scientists received lower Machiavellianism ratings than lobbyists. However, the professional affiliation did not affect the other trustworthiness measures, and it did not affect the perceived credibility of the provided information. Finally, we hypothesized that working for a university in combination with using an enthusiastic language style would result in especially high trustworthiness and credibility ratings. Although the results did reveal an interaction between language style and professional affiliation, it was in the opposite direction: Contrary to our hypotheses, we found that when the forum post author was a scientist, enthusiastic language led to lower integrity ratings and lower benevolence ratings than neutral language.

Overall, even though it was not hypothesized, the results show that the enthusiastic language style decreased the trustworthiness of the forum post author and the credibility of his information. One possible reason for this finding, derived from communication accommodation theory [46], might be the question-answer setting in which the information exchange took place. In the experiment, the help seeker asked for advice and formulated her question in a neutral language style. Therefore, the participants might have expected an answer in an equally neutral language style and perceived the enthusiastic language style as a violation to the introduced communication rule.

Another surprising finding is that the professional affiliation of the forum post author only affected the Machiavellianism measure because previous research has found that scientists are typically perceived as benevolent, sincere, and likable [20]. One reason for this finding might be that the author mentioned his professional affiliation just briefly at the beginning of his forum post. Hence, the manipulation might have been relatively weak.

The last unexpected finding was that scientists who used an enthusiastic language style got especially low integrity and benevolence ratings. One possible reason for this finding might be that scientists are typically perceived as being rational and objective and that this stereotypical image is not compatible with an enthusiastic language style. Lobbyists, on the other hand, might be perceived as people who relentlessly pursue a specific goal, and this stereotypical image might be more compatible with an enthusiastic language style.

### Limitations

Although the findings of this study highlight the importance of the language style and professional affiliation of people who are communicating health information in online settings, there are limitations to the generalizability of the results. One limitation could be the geographical location in which the experiment took place. More specifically, countries have developed different civic epistemologies (ways in which societies evaluate and discuss knowledge claims; see [47,48]). Discussions in Germany, for example, typically focus on “building communally crafted expert rationales, capable of supporting a policy consensus,” whereas in the United States, “information is typically generated by interested parties and tested in public through overt confrontation between opposing, interest‐laden points of view” [47]. Hence, information seekers in Germany may prefer neutral language styles as a constructive way of reaching a consensus. In the United States, however, informaiton seekers are used to emotionally laden discussions and therefore, may react differently to enthusiastic language styles. Another limitation could be the age of this study sample. Previous research has shown that age differences exist in regard to source monitoring and suggestibility to misinformation [49]. Young adults, for example, who grew up with modern information technologies and have been confronted with misinformation on the internet throughout their lives, may pay more attention to relevant source information when evaluating online information. Older adults, however, may not be as critical as younger adults because of their lack of experience with misinformation on the internet and therefore, pay less attention to relevant source information.

A further limitation concerns the topic of the forum posts and the chosen study sample. According to the Elaboration Likelihood Model of Persuasion [50], the personal relevance of a topic influences information processing: If a topic is personally relevant, peripheral cues become less important. Therefore, participants who do not considere the topic to be personally relevant may rely more heavily on peripheral cues such as the professional affilation of the forum post author when making trustworthiness and credibility judgments. Hence, to assess the generalizability of the results, future research needs to replicate this study in different communication settings and with different populations.

### Conclusions

When health information seekers are confronted with enthusiastic language in online forums, they may judge the information source as less trustworthy (especially when the information source is a scientist) and deem the communicated information less credible. Furthermore, health information seekers may perceive an information source as more trustworthy when he or she works for a university rather than a lobbying organization. These findings illustrate that health information seekers do not just react to health information on its own. In addition, they are also sensitive to the ways in which health information is presented (“Which language style is used to communicate health information?”) and who presents it (“Who does the health information source work for?”).

## References

[1] Higgins O, Sixsmith J, Barry MM, Domegan C (2011). A Literature Review on Health Information-Seeking Behaviour on The Web: A Health Consumer and Health Professional Perspective.

[2] Fox S, Duggan M (2013). Pew Research Center.

[3] Miles J, Petrie C, Steel M (2000). Slimming on the internet. J R Soc Med.

[4] Keelan J, Pavri-Garcia V, Tomlinson G, Wilson K (2007). YouTube as a source of information on immunization: a content analysis. J Am Med Assoc.

[5] Pandey A, Patni N, Singh M, Sood A, Singh G (2010). YouTube as a source of information on the H1N1 influenza pandemic. Am J Prev Med.

[6] Lewandowsky S, Ecker UK, Seifert CM, Schwarz N, Cook J (2012). Misinformation and its correction: continued influence and successful debiasing. Psychol Sci Public Interest.

[7] Metzger MJ, Flanagin AJ, Eyal K, Lemus DR, Mccann RM (2003). Credibility for the 21st century: integrating perspectives on source, message, and media credibility in the contemporary media environment. Ann Int Commun Assoc.

[8] Stadtler M, Bromme R, Rapp DN, Braasch JL (2014). The content–source integration model: a taxonomic description of how readers comprehend conflicting scientific information. Processing Inaccurate Information: Theoretical and Applied Perspectives from Cognitive Science and the Educational Sciences.

[9] Bromme R, Goldman SR (2014). The public's bounded understanding of science. Educ Psychol.

[10] Bromme R, Thomm E (2016). Knowing who knows: laypersons' capabilities to judge experts' pertinence for science topics. Cogn Sci.

[11] Keil FC, Stein C, Webb L, Billings VD, Rozenblit L (2008). Discerning the division of cognitive labor: an emerging understanding of how knowledge is clustered in other minds. Cogn Sci.

[12] Bromme R, Jucks R, Schober MF, Rapp DN, Britt MA (2017). Discourse and expertise: the challenge of mutual understanding between experts and laypeople. The Routledge Handbook of Discourse Processes.

[13] Bromme R, Kienhues D, Porsch T, Bendixen LD, Feucht FC (2010). Who knows what and who can we believe? Epistemological beliefs are beliefs about knowledge (mostly) to be attained from others. Personal Epistemology in the Classroom: Theory, Research, and Implications for Practice.

[14] König L, Jucks R (2019). When do information seekers trust scientific information? Insights from recipients’ evaluations of online video lectures. Int J Educ Technol High Educ.

[15] Choi W, Stvilia B (2015). Web credibility assessment: conceptualization, operationalization, variability, and models. J Assoc Inf Sci Technol.

[16] Metzger MJ, Flanagin AJ, Sundar SS (2015). Psychological approaches to credibility assessment online. The Handbook of the Psychology of Communication Technology.

[17] Pornpitakpan C (2004). The persuasiveness of source credibility: a critical review of five decades' evidence. J Appl Soc Psychol.

[18] Thon FM, Jucks R (2017). Believing in expertise: how authors' credentials and language use influence the credibility of online health information. Health Commun.

[19] Jucks R, Linnemann GA, Thon FM, Zimmermann M, Blöbaum B (2016). Trust the words: insights into the role of language in trust building in a digitalized world. Trust and Communication in a Digitized World.

[20] König L, Jucks R (2019). Hot topics in science communication: aggressive language decreases trustworthiness and credibility in scientific debates. Public Underst Sci.

[21] Zimmermann M, Jucks R (2018). How experts' use of medical technical jargon in different types of online health forums affects perceived information credibility: randomized experiment with laypersons. J Med Internet Res.

[22] Mayweg-Paus E, Jucks R (2015). Evident or doubtful? How lexical hints in written information influence laypersons' understanding of influenza. Psychol Health Med.

[23] Feinkohl I, Flemming D, Cress U, Kimmerle J (2016). The impact of personality factors and preceding user comments on the processing of research findings on deep brain stimulation: a randomized controlled experiment in a simulated online forum. J Med Internet Res.

[24] Flemming D, Cress U, Kimmerle J (2017). Processing the scientific tentativeness of medical research: an experimental study on the effects of research news and user comments in online media. Sci Commun.

[25] Bientzle M, Griewatz J, Kimmerle J, Küppers J, Cress U, Lammerding-Koeppel M (2015). Impact of scientific versus emotional wording of patient questions on doctor-patient communication in an internet forum: a randomized controlled experiment with medical students. J Med Internet Res.

[26] Flemming D, Cress U, Kimmig S, Brandt M, Kimmerle J (2018). Emotionalization in science communication: the impact of narratives and visual representations on knowledge gain and risk perception. Front Commun.

[27] Ghose A, Ipeirotis PG, Sundararajan A, Zaenen A, van den Bosch A (2007). Opinion mining using econometrics?: a case study on reputation systems. Proceedings of the 45th Annual Meeting of the Association of Computational Linguistics.

[28] Barry C, Hogan M, Torres AM, Vogel D, Guo X, Linger H, Barry C, Lang M, Schneider C (2016). Framing or gaming? Constructing a study to explore the impact of option presentation on consumers. Transforming Healthcare Through Information Systems.

[29] Starling S (2017). Evolutionary genetics: fantastic beasts - cephalopod RNA recoding. Nat Rev Genet.

[30] Brownlee C, Heald R (2018). The incredible shrinking spindle. Dev Cell.

[31] Keller MM, Hoy AW, Goetz T, Frenzel AC (2015). Teacher enthusiasm: reviewing and redefining a complex construct. Educ Psychol Rev.

[32] Frenzel AC, Goetz T, Lüdtke O, Pekrun R, Sutton RE (2009). Emotional transmission in the classroom: exploring the relationship between teacher and student enjoyment. J Educ Psychol.

[33] Keller MM, Goetz T, Becker ES, Morger V, Hensley L (2014). Feeling and showing: a new conceptualization of dispositional teacher enthusiasm and its relation to students' interest. Learn Instr.

[34] Kunter M, Klusmann U, Baumert J, Richter D, Voss T, Hachfeld A (2013). Professional competence of teachers: effects on instructional quality and student development. J Educ Psychol.

[35] Patrick BC, Hisley J, Kempler T (2000). 'What's everybody so excited about?': the effects of teacher enthusiasm on student intrinsic motivation and vitality. J Exp Educ.

[36] Burgoon M, Pauls V, Roberts DL, Dillard JP, Pfau M (2002). Language expectancy theory. The Persuasion Handbook: Developments in Theory and Practice.

[37] Barney LJ, Griffiths KM, Banfield MA (2011). Explicit and implicit information needs of people with depression: a qualitative investigation of problems reported on an online depression support forum. BMC Psychiatry.

[38] Bhamrah G, Ahmad S, NiMhurchadha S (2015). Internet discussion forums, an information and support resource for orthognathic patients. Am J Orthod Dentofacial Orthop.

[39] Kendal S, Kirk S, Elvey R, Catchpole R, Pryjmachuk S (2017). How a moderated online discussion forum facilitates support for young people with eating disorders. Health Expect.

[40] Sinha A, Porter T, Wilson A (2018). The use of online health forums by patients with chronic cough: qualitative study. J Med Internet Res.

[41] Wen H, Shi J, Zhang Y, Lu KH, Cao J, Liu Z (2018). Neural encoding and decoding with deep learning for dynamic natural vision. Cereb Cortex.

[42] Appelman A, Sundar SS (2015). Measuring message credibility: construction and validation of an exclusive scale. Journal Mass Commun Q.

[43] Küfner AC, Dufner M, Back MD (2015). The dirty dozen and the wicked nine: short scales for the detection of narcissism, machiavellism and psychopathy. Diagnostica.

[44] Jonason PK, Webster GD (2010). The dirty dozen: a concise measure of the dark triad. Psychol Assess.

[45] Hendriks F, Kienhues D, Bromme R (2015). Measuring laypeople's trust in experts in a digital age: the Muenster Epistemic Trustworthiness Inventory (METI). PLoS One.

[46] Dragojevic M, Gasiorek J, Giles H, Berger CR, Roloff ME (2015). Communication accommodation theory. The International Encyclopedia of Interpersonal Communication. First Edition.

[47] Jasanoff S, Dryzek JS, Norgaard RB, Schlosberg D (2011). Cosmopolitan knowledge: climate science and global civic epistemology. The Oxford Handbook of Climate Change and Society.

[48] Jasanoff S (2005). Designs on Nature: Science and Democracy in Europe and the United States.

[49] Mitchell KJ, Johnson MK, Mather M (2002). Source monitoring and suggestibility to misinformation: adult age-related differences. Appl Cogn Psychol.

[50] Petty RE, Cacioppo JT (1986). The elaboration likelihood model of persuasion. Adv Exp Soc Psychol.

